# Neutral is not fair enough: testing the efficiency of different language gender-fair strategies

**DOI:** 10.3389/fpsyg.2023.1256779

**Published:** 2023-09-29

**Authors:** Elsa Spinelli, Jean-Pierre Chevrot, Léo Varnet

**Affiliations:** ^1^Laboratoire de Psychologie et NeuroCognition, Université Grenoble Alpes, CNRS, Grenoble, France; ^2^Laboratoire Lidilem, Université Grenoble Alpes, Grenoble, France; ^3^Laboratoire des Systèmes Perceptifs, Département d'Études Cognitives, École Normale Supérieure, Université PSL, CNRS, Paris, France

**Keywords:** gender-fair language, generic masculine, gender-neutral noun, gender representation, masculine bias

## Abstract

In many languages with grammatical gender, the use of masculine forms as a generic reference has been associated with a bias favoring masculine-specific representations. This article examines the efficiency of gender-fair forms, specifically gender-unmarked forms (neutralization strategy, e.g., “l'enfant”) and contracted double forms (re-feminization strategy, e.g., “un·e enfant”), in reducing gender biases in language. Extensive empirical research has shown that gender-fair forms have the potential to promote more gender-balanced representations. However, the relative efficiency of these strategies remains a subject of debate in the scientific literature. In order to explore these questions, two experiments were conducted in French. We analyzed the response times and percent correct scores using a sentence evaluation paradigm, where the participants had to decide whether a second sentence starting with a gendered personal pronoun (“il” or “elle”) was a sensible continuation of the first sentence written in a gender-fair form. Experiment 1 confirmed that gender-unmarked forms are not fully effective in neutralizing the masculine bias. In Experiment 2, a comparison was made between gender-unmarked forms and contracted double forms, to assess their respective abilities to generate more balanced representations. The findings indicated that contracted double forms are more effective in promoting gender balance compared to gender-unmarked forms. This study contributes to the existing scientific literature by shedding light on the relative efficiency of neutralization and re-feminization strategies in reducing gender biases in language. These results have implications for informing efforts to promote more inclusive and unbiased language practices.

## 1. Introduction

In languages with grammatical gender, such as French, the use of masculine forms to refer to both males and females, as well as unspecified or mixed-gender groups, is a common linguistic convention (Gygax et al., 2019). As a result, while the feminine form always has a specific interpretation, the masculine form can be used in both a specific and a generic sense. For instance, in French, the word é*tudiantes* [students_*FEM*_] unambiguously refers to a group of female students, while the word é*tudiants* [students_*MASC*_] can refer to a group of only male students, both male and female students, or even students whose gender is unknown. This ambiguity poses challenges for language processing as the cognitive system needs to resolve the reference of the noun. This is evident in the following example, which is particularly challenging for French speakers due to the alternation of generic and specific senses of the same masculine form within a single sentence^1^:

*J'ai trente-quatre étudiants en cours, dont seulement sept sont des étudiants*

[I have thirty-four students_*MASC*_ in my class, only seven of whom are students_*MASC*_]

The resolution of this kind of ambiguity relies on various cognitive mechanisms, including world knowledge, context, and pragmatic cues. However, previous research suggests that the ambiguity is typically resolved to the disadvantage of the feminine gender, with the masculine generic favoring the activation of masculine-specific representations. For instance, in Experiment 1 of Gygax and Gabriel (2008), French-speaking participants had to decide, as fast as possible, whether the person referred to by a kinship term (e.g., *un frère* [a brother] or *une sœur* [a sister]) could be part of a group described by a role noun in the masculine generic form (e.g., *musiciens* [musicians_*MASC*_]). The results revealed a strong bias toward male kinship terms, indicating that male kinship terms were more readily associated with role nouns written in the masculine form. Similarly, when asked to estimate the percentage of women in a group described by a generic masculine term, participants consistently selected gender ratios below 50%, despite the role nouns being non-stereotyped in terms of gender (Tibblin et al., 2023). Moreover, studies have shown that the use of generic masculine language in questionnaires can impact the self-reported efficacy of female respondents (Chatard-Pannetier et al., 2005; Vainapel et al., 2015). Wilson and Ng (1988) used a priming paradigm to manipulate the perception of gender in ambiguous photographs through short sentences presented beforehand. The results indicated that the use of masculine generic language in the sentences biased perception toward male faces. Together, the findings from these experiments, as well as other studies mentioned below, provide robust evidence supporting the notion that the use of masculine-generic forms automatically activates masculine-specific representations.

One effective approach to reduce the influence of the masculine bias caused by masculine-generic forms is to employ gender-fair forms. Two main strategies can be distinguished regarding gender-fair language (see, e.g., Tibblin et al., 2023, Figure 1): neutralization on the one hand, which can be achieved, for instance, through the use of gender-unmarked words, and re-feminisation on the other hand, exemplified by the contracted double form. Gender-unmarked words have the same form for both male and for female referents,^2^ and are only differentiated by the preceding article. For instance, in French, *le ministre* vs. *la ministre* [the_*MASC*_ minister_*unmarked*_ vs. the_*FEM*_ minister_*unmarked*_] refer to a male or female minister respectively. In certain cases, gender information can be entirely removed: when using plural forms (*les ministres* [the_*plur,unmarked*_ ministers_*plur,unmarked*_]) or when an elision occurs on the article due to the phonological context (*le* or *la* replaced by *l'* if the following word begins with a vowel). For instance, *l'acrobate* [the_*unmarked*_ acrobat_*unmarked*_] can refer to either a female or a male acrobat. On the other hand, the re-feminisation strategy consists in explicitly listing the masculine and feminine forms, such as *un ou une ministre* [a_*MASC*_ or a_*FEM*_ minister_*unmarked*_]. This expression can be sometimes shortened into a contracted double form using a mid-dot (e.g., *un*·*e ministre*).^3^

**Figure 1:**
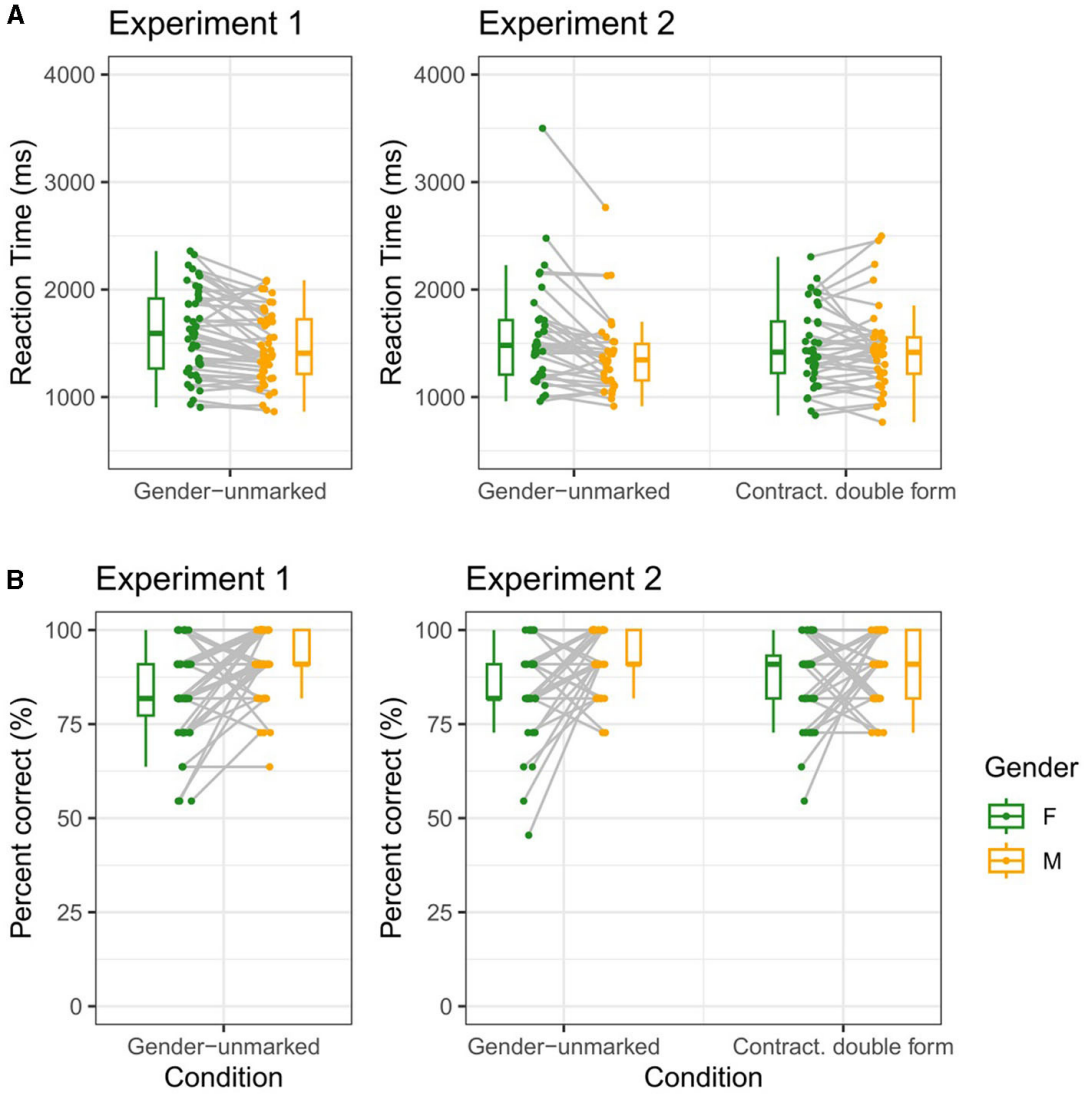
Average reaction times **(A)** and percentage of correct responses **(B)** for each participant in each of the two experiments. The boxplots show the median, and the 25^th^ and 75^th^ percentiles.

There are two important distinctions between the gender-unmarked form and the contracted double form. First, while the gender-unmarked form is truly neutral in the sense that it lacks any explicit gender markings, the contracted double form explicitly includes both the feminine and the masculine forms. Secondly, these two forms hold different sociolinguistic status. Gender-unmarked forms are relatively common in standard French, even in a generic masculine context, whereas contracted double forms are still socially marked: although their use has been promoted in France since 2015 by the *Haut Conseil à l'Égalité entre les femmes et les hommes* (Guide pratique pour une communication publique sans stéréotypes de sexe, 2022), and is occasionally found in official documents, they are still considered as a radical linguistic innovation and have been the subject of intense public and political debates in the French society (see Candea and Véron, 2019; Loison-Leruste et al., 2020 for more sociocultural context).

Extensive empirical research has been conducted to investigate whether the masculine bias associated with masculine generic forms can be eliminated or reduced through the use of gender-fair forms. Brauer and Landry (2008) conducted five studies comparing the activation of masculine representations in a masculine-generic context and in a double-form context in French. The results of all five experiments consistently demonstrated that double forms promote more gender-balanced representations. For instance, in the first experiment, participants cited nearly three times as many woman as a potential Prime Minister when the question was asked using a double form (*candidat/candidate* [candidate_*MASC*_/candidate_*FEM*_]), as compared to a generic masculine form (*candidat* [candidate_*MASC*_]) (Brauer and Landry 2008, exp. 1; see also Stahlberg and Sczesny, 2001). In a subsequent experiment, the use of masculine generics, as opposed to double forms, decreased the likelihood of participants naming a woman when asked to list their favorite heroes and artists (Brauer and Landry 2008, exp. 2; see also Stahlberg et al. 2001). The experimenters then conducted two experiments on adults and children, showing that grammatical form also influenced the gender of the imagined prototypical person when envisioning an occupational group (Brauer and Landry, 2008, exp. 3 and exp. 4). In a final experiment, participants read a short text about a professional gathering and estimated the percentages of men and women present. The findings indicated that the double form resulted in a more gender-balanced representation of the group when no information about its gender composition was provided (Brauer and Landry 2008, exp. 5; see also Braun et al., 1998, 2005; Xiao et al., 2023).

Studies investigating the efficiency of the neutralization strategy in reducing masculine bias are more seldom. Kim et al. (2023) conducted a study in which participants had to judge whether an individual, identified solely by a gendered first name, could be part of a group described either in a plural generic masculine form or a gender-neutral form. The results demonstrated that the use of gender-neutral forms dramatically reduced the male bias observed with masculine forms, and that both grammatical forms used equal cognitive resources, as indicated by comparable response times. Richy and Burnett (2021) conducted a gender judgement task that yielded similar findings. Non-gender-stereotyped role names introduced with a masculine article (e.g., *le journaliste* [the_*MASC*_ journalist]) were rated as masculine to a much higher degree than those introduced with a feminine article (e.g., *la journaliste* [the_*FEM*_ journalist]). In contrast, when the gender marking was completely eliminated, such as in *l'imperturbable journaliste* [the_*unmarked*_ unflappable journalist], the rating was close to balance (46%). These findings provide further support for the efficacy of gender-neutral forms in reducing masculine bias.

This raises the question of whether one of the two strategies (neutralization or re-feminization) is more effective in promoting gender-balanced representations. Only a few studies have been designed to address this question, and the conclusions drawn from them are generally inconsistent. Lindqvist et al. (2019) explored whether job candidates described in short paragraphs were perceived as more masculine or more feminine when the summaries were written using gender-neutral words (such as “the applicant” or the singular “they”) or a double pronoun (“he/she”). The results indicate that double forms produced more balanced gender representations compared to gender-neutral forms, both in English and in Swedish. These findings align with other experiments conducted in French and German, suggesting that the specific type of gender-fair form may have a differential effects, particularly when they interact with stereotypical information (Irmen and Roßberg, 2004; Braun et al., 2005; Irmen, 2007; Brauer and Landry, 2008). However, these conclusions contradict those of other researchers. In a recent study conducted in French, Tibblin et al. (2023) compared the perceived gender ratio within groups described using a single role noun in the masculine generic form or four different gender-fair forms. The results indicated that all tested gender-fair forms were equally successful in countering the male bias induced by the masculine form, resulting in estimated percentages of women ranging between 49.1 to 50.0%. Similar results have also been reported in a study by Stahlberg et al. (2001) investigating neutralization and re-feminization strategies in German. Furthermore, several studies have found that gender-neutral forms were able to largely eliminate the masculine bias (see, in particular, Richy and Burnett, 2021; Kim et al., 2023, in French). These discrepancies may be attributed to methodological issues, such as inadequate control for response biases or insufficiently precise measures that might have hindered the detection of potential effects. For example, Tibblin et al. (2023) demonstrated that the direction of the response scale in a gender ratio estimation paradigm has a systematic effect on the results.

In summary, both gender-unmarked forms (neutralization strategy) and contracted double forms (re-feminization strategy) have potential in reducing gender biases in language. However, gender-unmarked forms seem to yield intermediate results between double forms and masculine generic forms, suggesting a more nuanced impact on mental representations. Thus, the objective of the present study was twofold: firstly, to provide confirmation, using French sentences, that gender-unmarked forms are not fully effective in neutralizing the inherent masculine bias (Experiment 1); second, to compare this condition with a re-feminization strategy, specifically the contracted double form using a mid-dot (Experiment 2). Our hypothesis for the second experiment was that contracted double forms would yield more balanced representations than gender-unmarked forms.

As noted above, the choice of a particular methodology is crucial for detecting potential differences among gender-fair forms. To ensure precise measurements capable of capturing these distinctions, we modified the sentence evaluation paradigm employed in prior studies (Gygax et al., 2008; Garnham et al., 2012). Compared to the more commonly-used gender-ratio estimation experiment, the sentence evaluation paradigm offers several advantages. It is less prone to ceiling effects as it relies on response time measurements. Additionaly, it is less susceptible to criterion effects, by avoiding subjective scaling task. Finally, stereotypes associated with nouns and contexts are known to greatly contribute to gendered interpretations. Thus, it is crucial to carefully control for this factor in experiments aimed at assessing the influence of grammatical gender. In practice, experimenters usually evaluate the level of stereotype associated with their stimuli prior to testing, in order to establish a stereotype-neutral condition (Irmen and Roßberg, 2004; Xiao et al., 2023). We adopted a similar approach in the present study.

## 2. Experiment 1

### 2.1. Methods

#### 2.1.1. Pre-test and stimuli selection

The pre-test consisted in judging the extent to which a role noun was more represented by men or women. A list of 46 gender-unmarked words beginning with a vowel was tested (see Supplementary Table S1). A total of 74 participants (16 men and 58 women) took part in the pretest. They were all second-year psychology students recruited on a voluntary basis. Participants were asked to judge whether each word (e.g., *accordéoniste* [accordionist]) was more likely to be represented by women or men. They responded by ticking a line going from 100% men at the left edge of the line to 100% women at the right edge of the line (counterbalanced across participants). Then, masculine percentage was calculated for each word (for example, if a word received a 60% feminine rating, it was considered 40% masculine). The average masculine ratings across all participants are presented in Supplementary Table S1. The stereotypicality estimate were in close agreement with those obtained by Misersky et al. (2014), as evidence by a strong correlation in the masculine rating scores for the item examined in both their study and the present one (Supplementary Figure S1).

We wanted our stimuli to be as unstereotyped as possible. Thus, we first selected the 25 words that came closest to 50% masculine. We further excluded two words (é*quilibriste* [tightrope walker] and *astrologue* [astrologer]) that had a high standard deviation (19.8 and 24.58 respectively). Another one (*harmoniciste* [harmonica player]) was also removed, as over 10% of our participants did not know the meaning of this word and did not respond to this item. The 22 remaining words were then selected for the experiment (means ranged from 45.74 to 59.78% and standard deviations ranged from 6.724 to 17.907).

#### 2.1.2. Participants

Forty-seven psychology students (44 women, 3 men) at Grenoble-Alpes University, participated in this study for course credit. They were all French native speakers and had either normal or corrected vision.

#### 2.1.3. Stimuli

Each of the 22 gender-unmarked, non-stereotyped words that were selected by the means of the pretest (e.g., é*lève*, [student]), was presented in a carrier sentence (hereafter context sentence). The gender-unmarked words all began with a vowel, so that the determiner *l'* [the_*unmarked*_] could be used, thus enabling grammatical gender neutrality in the context sentences (e.g., *L'élève commenait à réviser*, [The_*unmarked*_ student_*unmarked*_ began to study]). Twenty-two continuation sentences beginning by *il* [he] or *elle* [she], were associated to these context sentences and constituted a good continuation (e.g., *Elle passait l'examen dans 15 jours* [She was taking the exam in 15 days]). The list of all context and continuation sentences for the selected items is presented in Supplementary Table S2. There were also 22 gender-unmarked words beginning with a vowel (e.g., *esclave*, [slave]) presented in context sentences (e.g., *L'esclave essaya de s'enfuir* [The slave attempted to escape]) and followed by continuation sentences that constituted a bad continuation (e.g., *Il faisait ses courses de Noël*, [He was doing his Christmas shopping]). Moreover, there were also 22 consonant-initial filler words presented in context sentences and followed by continuation sentences that constituted a good continuation (e.g., *Les vendeurs ont fait un bon chiffre. Ils ont atteint les dix-mille euros*. [The sellers sold very well. They had takings of ten thousand euros.]). There were also 22 consonant-initial filler words presented in context sentences and followed by continuation sentences that constituted a bad continuation (e.g., *Le lion est le roi de la savane. Il doit aller chercher les enfants à l'école*. [The lion is king of the savannah. He has to pick up the kids from school.]). Consonant-initial filler words could be followed by either *le* [the_*MASC*_], *la* [the_*FEM*_] or *les* [the_*plural, unmarked*_] as determiners.

#### 2.1.4. Procedure

Participants were seated ~50 cm from a computer screen. They were informed that they would read a succession of sentences, in pairs, and that they would have to determine as quickly as possible whether the second sentence was a good or bad continuation of the first one. The experiment was run on E-Prime 2.0 software (Psychology Software Tools, Pittsburgh, PA). The first sentence (context sentence) remained on the screen for 3 s. It was then replaced by the second one (continuation sentence). The participant then had to respond as quickly as possible by using one of the two keys on the keyboard. They were asked to press the “yes” key with their preferred hand. The intertrial interval was 3 s. The experiment began by 10 practice trials followed by a list of 88 (66 fillers and 22 experimental) trials that were randomly presented.

For the 22 experimental trials, half of the continuation sentences began with *il* [he] and the other half with *elle* [she]. The gender assignment of the continuation sentence of each test item was counterbalanced in two experimental lists. The response of the participants to each stimulus were recorded, as well as their response time from the onset of the continuation sentence presentation.

#### 2.1.5. Analysis

Data were analyzed through Bayesian hierarchical mixed effects regression models. This choice was motivated by the need to examine the influence of the predictor variable, *grammatical gender*, while accounting for the random variation between participants and items. By including the factors *participant* and *item* as random intercepts, we could capture the baseline response times for each participant and item separately, allowing for a more precise estimation of the effect of grammatical gender.

Firstly, reaction times for all positive responses were examined.^4^ The *z*-scored response time data, *RTz*, was fitted with a model including a fixed effect of *grammatical gender* (0: feminine; 1: masculine) and two random effects of factors *participant* and *item*, entered as random intercepts:


(1) $$\begin{array}{l} RTz=\alpha_{p,i}+\beta_{gender}\cdot gender+\epsilon \end{array}$$

where ϵ~N(0,σ). Each pair of a specific participant *p* and a specific item *i* was associated with a unique intercept α_*p,i*_, whose distribution was described with three hyperparameters β_0_, σ_*participant*_ and σ_*item*_ capturing the variability of individual differences in response times:


(2) $$\begin{array}{l} \alpha_{0,p,i}=\beta_{0}+\beta_{participant}+\beta_{item} \end{array}$$

with $\beta_{participant}\sim N\left(0,\sigma_{participant}\right)$ and $\beta_{item}\sim N\left(0,\sigma_{item}\right)$. Weakly informative, conservative distributions $\beta_{X}\sim N\left(0,1\right)$ were used for all other effect-related parameter and for the hyperprior β_0_ (recall that all predictors were standardized). The dispersion of the participant-specific and item-specific parameters was associated with a prior distribution σ_*X*_~Gamma(2, 0.1), enforcing the pooling of information to improve estimation. The same prior was used for σ. A graphical summary of this model and the prior distributions used is provided in Supplementary Figure S2.

Secondly, participants' responses (yes/no) per item were analyzed using a logistic regression model with the same design as for the response time data. The probability of correct response was assumed to follow the general form for the psychometric function, with a chance level at 0.5:


(3) $$\begin{array}{l} P\left(\text{correct}\right)=0.5+\left(0.5-p_{miss}\right)\cdot p \end{array}$$

where *p*_*miss*_ represents the probability of lapse (defining the upper asymptote of the psychometric function) and *p* is a cumulative density function. This function is described with the standard logistic regression formula, following the same hierarchical model design as before:


(4) $$\begin{array}{l} logit\left(p\right)=\alpha_{p,i}+\beta_{gender}\cdot gender \end{array}$$

For simplicity, *p*_*miss*_ was assumed to be the same for all participants and was estimated from the data using a weak *Beta*(5, 10) prior.

All analyses were carried with R version 4.3.0 (R Core Team, 2020), using the RStan interface to Stan for Bayesian modeling (Carpenter et al., 2017; Stan Development Team, 2020). Seven chains of 7,000 samples each were run independently (3,000 burn-in samples, estimates based on 4,000 samples). Their convergence was monitored through standard summary statistics R-hat and Effective Sample Size, absence of divergent transition, and visual inspection of posterior distributions. Throughout this article, Bayesian estimates will be reported along with their 95% credible intervals, providing an assessment of the reliability of the results. All the codes and data supporting this analysis are openly available on Github at https://github.com/LeoVarnet/GenderFair/.

### 2.2. Results

**Figure 2:**
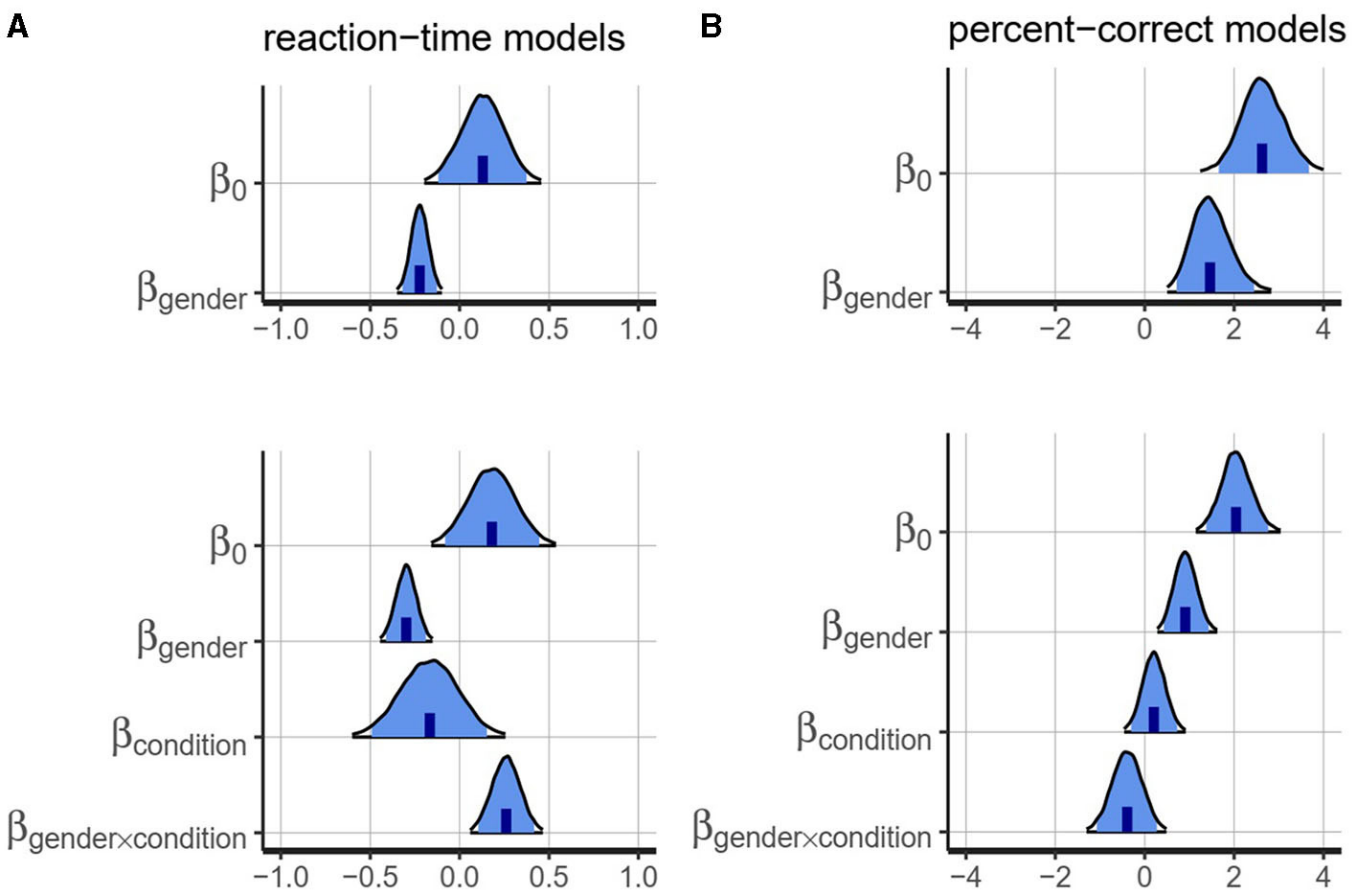
Posterior distributions for all fixed-effect parameters in the reaction-time models **(A)** and the percent-correct models **(B)**. Upper panels correspond to Experiment 1, lower panels to Experiment 2. The blue regions indicate 95% credible intervals. The black contours delimit the 99% credible interval.

The results from Experiment 1, averaged by participant, grammatical form and grammatical gender, are shown in Figure 1 (left panels). The posterior distributions for the intercept and fixed effects model parameters are shown in Figure 2 (upper panels). Posterior distributions for all parameters in the model are presented in Supplementary Figure S4.

#### 2.2.1. Positive response times

The average reaction times in the gender-neutral form condition was 1,594 ms ± 400 ms SD for continuation sentences beginning with a feminine pronoun and 1,468 ms ± 333 ms SD for continuation sentences beginning with a masculine pronoun. The posterior distribution for the parameter β_*gender*_ in the varying-intercept hierarchical model revealed a strong effect of the grammatical gender factor, with an estimate centered around −0.22, *CI*_95%_= (−0.32, −0.13).

It is important to note that reaction times varied noticeably across different items. For instance, the continuation sentence following *l'acrobate* [the acrobat] elicited a short average response time of 1,264 ms ± 313 ms (SD), while the one following *l'adulte* [the adult] required more processing time (mean response time = 1,845 ms ± 778 ms SD). These differences resulted in non-overlapping credible intervals for these items in the model (see Supplementary Figure S4).

#### 2.2.2. Percent correct scores

Participants achieved scores ranging from 55 to 100% for feminine continuation sentences (mean = 84%), and from 64 to 100% for masculine continuation sentences (mean = 91%). This difference was associated with a strong β_*gender*_ coefficient in the hierarchical logistic regression model [mean = 1.49, *CI*_95%_= (0.72, 2.45)]. The model also yielded a reliable estimate of *p*_*miss*_ to 6% [*CI*_95%_= (4%, 9%)].

Similar to response times, a strong variability was found in the mean performance achieved on each specific item. A separate multivariate Gaussian distribution fitted on the vectors of random intercepts from the two models identified that the two variables were likely negatively correlated [*R* = −0.43, *CI*_95%_= (−0.73, −0.05)], indicating that the words corresponding to longer response times also yielded more errors.

### 2.3. Interim discussion

Experiment 1 aimed to investigate the effects of grammatical gender on response times and accuracy in sentence processing, specifically in a context where the words were previously referred to with gender-unmarked forms. The context sentence did not provide any grammatical-gender cue, and the words were selected to be stereotype-neutral (e.g., *L'élève commenait à réviser* [The_*unmarked*_ student_*unmarked*_ began to revise.]). Based on previous investigation by Irmen and Roßberg (2004) and Irmen (2007), our hypothesis was that even in this neutral context, a masculine bias would persist, resulting in a disadvantage in processing continuation sentences beginning with a feminine pronoun (e.g., *Elle passait l'examen dans 15 jours* [She was taking the exam in 15 days.]).

The two statistical models supported our hypothesis, revealing a clear effect of the grammatical gender of the pronoun in the continuation sentence on both response times and accuracy scores. Specifically, sentences beginning with *elle* [she] required longer processing time and yielded higher error rates.

One critical question remains regarding the generality of this result. Does the observed masculine bias extend to all gender-fair forms, or is it specific to gender-neutral form? Our hypothesis was that the use of a re-feminization strategy could potentially eliminate the masculine bias. The rationale behind this hypothesis stems from the notion that re-feminized forms explicitly reintroduce grammatical gender markers associated with the feminine gender. Therefore, if the masculine bias is primarily driven by the absence of grammatical gender cues in gender-neutral forms, the re-feminized forms should counteract this bias.

In order to test this hypothesis, we designed a second experiment comparing the processing of gender-unmarked forms with contracted double forms. We expected that, in the second case, the observed masculine bias would be totally eliminated, or at least drastically reduced.

## 3. Experiment 2

### 3.1. Methods

#### 3.1.1. Participants

Seventy psychology students (64 women, 6 men) at Grenoble-Alpes University participated in this study for course credit. All subjects were native French speakers, with normal or corrected vision. Thirty-four participants were assigned to the gender-unmarked condition and 36 to the contracted double form condition.

#### 3.1.2. Stimuli

The gender-unmarked condition was a replication of experiment 1. Hence, the stimuli in this condition were the same as those of experiment 1, that is: context sentences began with *l'* and continuation sentences began with either *il* or *elle*. In the contracted double form condition however, the 22 gender-unmarked words presented in the context sentences were not preceded by the determiner *l'* [the_*unmarked*_] but by *un*·*e* [a_*MASC*·*FEM*_].^5^ Continuation sentences began with either *il* [he] or *elle* [she], as in experiment 1. For example one participant of the contracted double form condition would read *Un*·*e élève commenait à réviser* [A_*MASC*·*FEM*_ student_*unmarked*_ began to study], followed by *Elle passait l'examen dans 15 jours* [She was taking the exam in 15 days].

#### 3.1.3. Procedure

Participants were randomly assigned to either the gender-unmarked or the contracted double form condition. Apart from that, the procedure followed that of experiment 1.

### 3.2. Data analysis

The structure of the models in experiment 2 replicated that employed in experiment 1, with the only difference that the models also include the effect of factor *condition* (0: gender-unmarked; 1: contracted double) and a two-way interaction between this factor and *grammatical gender* (0: female; 1: male).

### 3.3. Results

The results from Experiment 2, averaged by participant, grammatical form and grammatical gender in each of the two conditions, are shown in Figure 1 (right panels). The posterior distributions for the intercept and fixed effects model parameters is shown in Figure 2. Posterior distributions for all parameters in the model are presented in Supplementary Figure S5. A *post-hoc* sensitivity analysis was also conducted on parameters β_*condition*_ and β_*gender*×*condition*_. The details and results are provided in the Supplementary material.

In the gender-unmarked condition, continuation sentences beginning with a feminine pronoun yielded a mean reaction time of 1,575 ms ± 505 SD and an average accuracy score of 84.2%. Conversely, continuation sentences beginning with a masculine pronoun had a mean reaction time of 1,394 ms ± 370 SD and an average score of 92.2%. These findings closely aligned with the results observed in Experiment 1. In particular, the posterior distributions for β_0_ and β_*gender*_ were highly consistent between the two experiments (recall that, because of the model design, β_*gender*_ codes for the difference between grammatical genders in the gender-unmarked condition).

In contrast, for the contracted double form condition, the measured reaction times were similar between feminine (mean = 1,467 ms ± 366 SD) and masculine continuation sentences (mean = 1,446 ms ± 395 SD). As a result, the posterior distribution for the β_*gender*×*condition*_ parameter in the response time model revealed a strong interaction effect [mean = 0.26, *CI*_95%_= (0.11, 0.42)]. There was no main effect of condition, as indicated by a posterior distribution for β_*condition*_ centered around 0 [mean = −0.17, *CI*_95%_= (−0.49, 0.14)], suggesting that gender-unmarked and contracted double forms led to similar processing times of the following continuation sentence, on average. For percent correct accuracy, the 95% credible interval for both β_*condition*_ and β_*gender*×*condition*_ overlapped with zero to a large extent [β_*condition*_: mean = 0.20, *CI*_95%_= (−0.31, 0.73); β_*gender*×*condition*_: mean = −0.39, *CI*_95%_= (−1.07, 0.29)]. It is however possible that this absence of noticeable impact of factor *condition* on percent correct scores was partly due to a ceiling effect, as the scores were quite high overall (86.4% for feminine pronoun, 90.2% for masculine pronoun).

Similar to Experiment 1, we measured the correlation between item-specific random intercepts from the reaction-time model and from the percent-correct model. No reliable correlation was found [*R* = 0.23, *CI*_95%_= (−0.19, 0.6)], again possibly because of a ceiling effect in the percent correct scores.

## 4. General discussion

The objective of the present study was to examine the efficiency of gender-unmarked forms and contracted double forms in mitigating gender biases in language. Two experiments were conducted, in which participants had to judge the consistency of a pair of French sentences: a context sentence where the gender of the character was intentionally left unspecified, using gender-fair forms, and a continuation sentence beginning with a feminine or a masculine singular pronoun. All nouns included in the experiment were pre-tested for the absence of strong gender stereotypes. In the first experiment, we confirmed that gender-unmarked forms in French were not fully successful in neutralizing the masculine bias. Specifically, sentences beginning with a feminine pronoun exhibited longer processing times and higher error rates compared to those beginning with a masculine pronoun, indicating the persistence of a masculine bias even in a gender-neutral context. For instance, following the context sentence *L'élève commençait à réviser* [The_*unmarked*_ student_*unmarked*_ began to revise], the feminine continuation sentence *Elle passait l'examen dans 15 jours* [She was taking the exam in 15 days] required more time to process and resulted in more errors compared to the masculine continuation sentence *Elle passait l'examen dans 15 jours* [She was taking the exam in 15 days]. In the second experiment, we contrasted gender-unmarked forms (such as *L'élève*) with contracted double forms using a mid-dot (such as *Un*·*e élève*). The results revealed that the use of contracted double forms, which reintroduce grammatical gender markers associated with the feminine gender, eliminated the gender bias observed in the gender-unmarked condition. In the contracted double form condition, there was no significant difference in processing time between feminine and masculine continuation sentences, suggesting that the re-feminization strategy successfully counteracted the masculine bias.

The results of Experiment 1 are consistent with our hypothesis: gender-unmarked forms in French are not fully effective in neutralizing masculine bias. This finding is in line with previous research demonstrating that neutral wordings elicit a male bias, in English and Swedish (Lindqvist et al., 2019) as well as in German (Irmen and Roßberg, 2004). However, it contrasts with several studies that found no bias induced by gender-neutral forms (e.g., Tibblin et al. 2023, Richy and Burnett 2021 and Kim et al. 2023 in French, Stahlberg et al. 2001 in German). It is possible that these studies failed to capture the difference in masculine bias due to methodological limitations. More specifically, some estimates approached ceiling levels, making it difficult to detect potential effects (e.g., Tibblin et al., 2023). Furthermore, in some experiments, participants may have been able to understand the study's objective and adjusted their responses accordingly. In contrast, we believe that the sentence evaluation paradigm used in the present study is less susceptible to ceiling effects and less prone to the development of conscious strategies by participants. Finally, differences in the test population demographics may also explain these discrepancies. This point will be discussed in section 4.3.

The observation of a masculine bias in the absence of any grammatical marker may seem surprising. At this point we can only speculate about the causes of this effect. Two possible, non-exclusive explanations are considered in the following paragraphs: a first one based on lexical frequency effects, and another one based on semantics.

### 4.1. Prevalence of gender-neutral nouns in the masculine form

An interesting explanation for the observed masculine bias is based on the fact that singular gender-neutral words, despite having no fixed grammatical gender, are often used as generic masculine forms. This is the case, for example, in sentences such as *Ils ont eu un enfant* [They had a_*MASC*_ child] or *Il faut que j'aille voir un orthopédiste* [I need to see an_*MASC*_ orthopedist]. These sentences employ gender-neutral nouns with a singular masculine marking of the pronoun to refer to individuals in a generic sense.

Because of the prevalence of these singular generic masculine constructions, gender-neutral words are more frequently seen in their masculine forms. Therefore, when presented with a gender-neutral form such as *L'enfant se roulait par terre en criant* [The_*unmarked*_ child was rolling on the floor screaming], the reader is more likely to interpret the noun *L'enfant* as a masculine. As a result, they may take more time to process the feminine continuation sentence *Elle faisait encore un caprice* [She was throwing another tantrum] than the masculine one, therefore giving rise to a masculine bias. In other words, even though the noun itself lacks explicit gender markings, readers would tend to default to a masculine representation based on the prevalent usage of gender-neutral words as generic masculine in the language.

### 4.2. Gender-specific interpretation of gender-neutral nouns

A similar effect can be conceived at a semantic level. As noted by Richy and Burnett (2021), formally gender-neutral terms are often used in ways which are covertly gendered. This is the case, for example, in the following English sentences (Black and Coward, 1981; Cameron, 1995):

*Young people should be out interfering with the local maidens*.

*We cannot tolerate attacks on the wife of an American citizen*.

Here, the generic nouns “people” and “citizen” are used to specifically refer to heterosexual men, thereby excluding women. Such usage suggests that English speakers tend to associate strong masculine representations with gender-neutral expressions. In French, Michard (1999) noted similar examples by studying the gendered discourse in the work of anthropologists, such as:

*Le village entier partit le lendemain dans une trentaine de pirogues, nous laissant seuls avec les femmes et les enfants dans les maisons abandonnées*. [The entire village left the next day in about thirty canoes, leaving us alone with the women and children in the abandoned houses.]

*Même si l'enfant est mort-né, les Baruya souponnent toujours leur femme d'avoir tué leur enfant*. [Even if the child is stillborn, the Baruya always suspect their wives of killing the child].

These examples highlight that the use of gender-neutral nouns does not necessarily imply inclusive reference. Instead, due to our androcentric experiences and cultural influences, gender-neutral nouns may carry underlying masculine stereotypes. This could in turn explain why, in the present study, the items resulted in more activation of the masculine representations.

### 4.3. Further studies

In order to disentangle the locus (lexico-statistics vs. semantic level) of the masculine bias in the absence of gender marking, we plan to run a series of experiments using stimuli than convey conflicting cues between frequency of usage and semantics. In French some generic nouns occur only in the feminine form (e.g., *une personne* [a_*FEM*_ person_*FEM*_], *une victime* [a_*FEM*_ victim_*FEM*_], *une recrue* [a_*FEM*_ recruit_*FEM*_]). In this case, the prevalence of the grammatical feminine form would conflict with the automatic activation of the masculine representations at the semantic level. If a masculine bias is still observed in noun phrases such as *l'honnête victime* [the_*unmarked*_ honest_*unmarked*_ victim_*FEM*_] it would suggest that the masculine bias is not lexically driven by usage frequency but by the automatic activation of the masculine representations at the semantic level. Interestingly, Brauer and Landry (2008)'s results (experiment 3) suggest that this is not the case: When participants were asked to imagine a prototypical human representation of a profession (by describing him/her, finding a name, attributing an age and a salary, etc.), more women were described when the instructions contained the feminine noun *personne* (e.g., *Essayez d'imaginer cette personne le plus vivement possible*, [try to imagine this_*FEM*_ person_*FEM*_ as vividly as possible]) than when the masculine noun *individu* was used (e.g., [try to imagine this_*MASC*_ individual_*MASC*_ as vividly as possible]).

Another promising direction for broadening the scope of the study would involve testing the presence of masculine bias using unbalanced stereotyped words, in order to examine how the observed effects interact with stereotypes. This line of investigation would serve two complementary purposes. First, it would enable us to validate the robustness of the effects measured in our controlled experimental conditions, when tested in more ecological situations. Second, this would facilitate the manipulation of the semantics toward feminine representations. This is particularly relevant considering that frequency of usage may favors masculine representations due to the masculine generic rule (see section 4.2). Should we observe a persistent masculine bias in sentences such as *l'hystérique hurlait* [the_*unmarked*_ hysterical person_*unmarked*_ was yelling] (where *hystérique* is strongly biased toward feminine representations), it would imply that the masculine bias is primarily driven by lexical frequency.

Finally, previous research has highlighted the influence of participant's gender and self-reported masculinity levels on gender processing effects (Casado et al., 2023). Our participant sample exhibits a marked gender imbalance (108 women vs. 9 men in total), reflecting the gender ratio typical of undergraduate psychology students at our University. It would be interesting to replicate the present experiment with a more balanced number of male and female participants to assess the potential influence of participant gender on the findings. Similarly, it is plausible that the effects described in this study are also conditioned by the age and socio-economic status of the participant. Indeed, as noted in the Introduction section, there is an important sociolinguistic distinction between the gender-unmarked form and the contracted double form in French. In this study, our participant cohort was highly homogeneous in terms of age and professional orientation. Therefore, further research endeavors should be undertaken to explore the impact of these variables on the relative effectiveness of neutralization and re-feminization strategies in mitigating gender biases in language processing. In any case, it is worth noting that the subpopulation under examination (mostly young female students) may reasonably be expected to exhibit *lower* susceptibility to gender biases in language compared to other demographic groups. Consequently the present findings might be considered as a conservative estimate of the effect that would be observed in a more representative sample of the general population.

## Data availability statement

All the codes and data supporting this study are openly available on Github at https://github.com/LeoVarnet/GenderFair/.

## Ethics statement

The studies involving humans were approved by Comité d'Ethique de la Recherche Paris-Descartes. The studies were conducted in accordance with the local legislation and institutional requirements. The participants provided their written informed consent to participate in this study.

## Author contributions

ES: Writing—review and editing, Data curation, Methodology, Resources. J-PC: Methodology, Writing—review and editing, Conceptualization. LV: Writing— review and editing, Formal analysis, Investigation, Writing—original draft.

## Footnotes

^1^The use of this sentence has been reported by Hofstadter and Sander (2013). The authors also stressed that its comprehension requires some mental effort. In practice French speakers would usually circumvent this paradoxical phrasing by opting for a more explicit wording such as *J'ai trente-quatre étudiants en cours, dont seulement sept sont des garçons* [I have thirty-four students in my class, only seven of whom are boys]. Nevertheless, it is important to note that the original sentence remains grammatically correct. It can therefore be seen as a thought-provoking example highlighting the challenges posed by plural masculine terms.

^2^We refrain from using the term “epicene words” (Tibblin et al., 2023) here as it sometimes also includes generic nouns such as *une personne* or un *individu* which can refer to both male or female objects, but have a fixed grammatical gender. Alternative terms used as synonyms of gender-unmarked include “common-gender” (Richy and Burnett, 2021) and “non-differentiating” (Braun et al., 2005).

^3^Again, there is some disagreement on the terminology, as some authors use the term “gender-neutral” (Brauer and Landry, 2008; Vainapel et al., 2015) or “gender-balanced” (Gygax et al., 2008). We decided to stick with the terms used by Tibblin et al. (2023).

^4^A confirmatory analysis excluding reaction time data larger/smaller than the mean ± 2 SD for each participant yielded essentially the same results.

^5^When it comes to inclusive writing, there are several options in French. In our day-to-day reading, we may encounter hyphens (un-e), parentheses (un(e)) or the mid-dot (un·e), as there are no official characters so far. In this experiment, we chose to use the mid-dot. The mid-dot has the advantage of having no other function in the French language, unlike hyphens or parentheses, which are used to link terms together or to add precision to a written text.
