# Supplementary material for: Neutral is not fair enough: testing the efficiency of different language gender-fair strategies
Source: Front Psychol. 2023 Sep 29;14:1256779. doi: 10.3389/fpsyg.2023.1256779 (PMC10570613; doi:10.3389/fpsyg.2023.1256779)
Supplement: Supplementary file 1 [file Data_Sheet_1.pdf]

Supplementary Material

Supplementary materials for the article “Neutral is not fair enough: testing the efficiency of different language gender-fair strategies” (Elsa Spinelli, Jean-Pierre Chevrot, Léo Varnet). Corresponding author: Léo Varnet (leo.varnet@cnrs.fr). All the codes and data supporting this study are openly available on Github at `https://github.com/LeoVarnet/GenderFair/`

1 ITEMS AND STIMULI

1.1 Pilot experiment

Table S1 presents the list of gender-unmarked words tested in the pilot experiment (see section Pre-test and stimuli selection of the article). Participants were asked to judge whether each word was more likely to be represented by women or men. The average masculine percentage and the standard deviation for each word are indicated in the second column.

The measured masculinity scores were compared with the results from Misersky et al. (2014). Of the 46 role nouns tested in the present study, 14 were also tested by Misersky et al. The correlation between the masculinity judgment in the two study was very high: r(12) = 0.9087, $p < 0.001$ (see Supplementary Figure S1).

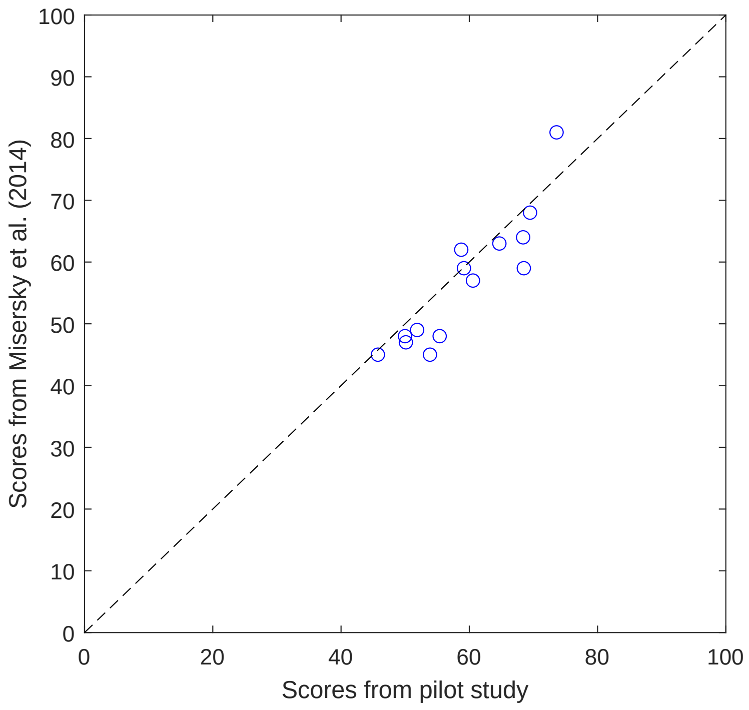

Figure S1. Relationship between the scores measured in the present study and the ones from Misersky et al. (2014).

Anarchiste	67% ± 14 S.D.
Œnologue	69% ± 17 S.D.
Équilibriste	53% ± 20 S.D.
**Architecte**	59% ± 13 S.D.
**Élève**	50% ± 10 S.D.
**Aveugle**	56% ± 13 S.D.
**Otage**	50% ± 18 S.D.
**Athlète**	60% ± 14 S.D.
Humoriste	61% ± 16 S.D.
Aristocrate	67% ± 18 S.D.
Imbécile	71% ± 17 S.D.
Accordéoniste	64% ± 20 S.D.
Esclave	61% ± 19 S.D.
Adversaire	60% ± 16 S.D.
**Adulte**	51% ± 7 S.D.
**Optimiste**	46% ± 17 S.D.
Alcoolique	68% ± 14 S.D.
**Enfant**	52% ± 10 S.D.
Archéologue	61% ± 15 S.D.
Ivrogne	76% ± 14 S.D.
**Interprète**	46% ± 17 S.D.
**Obèse**	57% ± 13 S.D.
**Urbaniste**	59% ± 15 S.D.
**Orthopédiste**	51% ± 17 S.D.
Automobiliste	65% ± 17 S.D.
Arbitre	74% ± 16 S.D.
Urgentiste	60% ± 16 S.D.
Autiste	61% ± 15 S.D.
Orthophoniste	40% ± 19 S.D.
Hystérique	29% ± 17 S.D.
**Ergonome**	52% ± 17 S.D.
Harmoniciste	53% ± 15 S.D.
**Analphabète**	56% ± 12 S.D.
Ébéniste	74% ± 14 S.D.
**Amnésique**	50% ± 12 S.D.
**Indigène**	59% ± 13 S.D.
**Artiste**	50% ± 11 S.D.
**Infirme**	56% ± 10 S.D.
**Acrobate**	54% ± 17 S.D.
Ornithologue	60% ± 14 S.D.
Alpiniste	70% ± 14 S.D.
**Interne**	53% ± 16 S.D.
**Ancêtre**	56% ± 15 S.D.
**Albinos**	60% ± 14 S.D.
Aubergiste	61% ± 20 S.D.
Astrologue	55% ± 25 S.D.

Table S1. List of all role nouns tested in the pilot experiment, with the corresponding average masculine rating (± standard deviation). The selected words are indicated in bold font.

1.2 Experiments 1 and 2

Table S2 presents the list of context and continuation sentences used in experiments 1 and 2. This particular wording correspond to the condition *Masculine* and *Gender-unmarked*. Note that these 22 "test" stimuli were shuffled with 44 "distracting" stimuli (see section 2.1.3).

	Context sentence	Continuation sentence
1	L'albinos ne pouvait pas rester longtemps dehors.	Il devait vite rentrer dans une pièce sombre.
2	L'amnésique avait encore tout oublié.	Il n'était pas près de guérir.
3	L'ancêtre ne veut pas aller aux urgences.	Il a renoncé à se soigner.
4	L'enfant se roulait par terre en criant.	Il faisait un encore caprice.
5	L'ergonome se mit à travailler.	Il voulait finir rapidement.
6	L'indigène arrive en costume de fête.	Il se marie aujourd'hui.
7	L'infirme boîte et tombe souvent.	Il semble désespéré.
8	L'optimiste se doutait avoir gagné.	Il était vraiment très chanceux.
9	L'orthopédiste prescrit oralement en plus de l'écrit.	Il savait son patient dyslexique.
10	L'otage ne mangeait pas depuis 10 jours.	Il avait perdu beaucoup de poids.
11	L'artiste finissait tout juste de peindre.	Il était prêt à vendre la toile.
12	L'adulte était en train de voter.	Il avait choisi son maire.
13	L'analphabète a réussi à conjuguer au futur.	Il est fier d'avoir fait des progrès.
14	L'architecte surveille les travaux en cours.	Il pense qu'il faudra deux mois de plus.
15	L'athlète termine en passant devant tous les autres.	Il s'effondre ensuite de fatigue.
16	L'aveugle veut vivre de façon autonome.	Il fait ses courses sans aide.
17	L'élève commençait à réviser.	Il passait l'examen dans 15 jours.
18	L'interne surveillait attentivement.	Il ne voulait pas de bruit pendant l'opération.
19	L'interprète continua de traduire.	Il aimait beaucoup l'auteur.
20	L'obèse ne bouge pas suffisamment.	Il ne perd pas de poids.
21	L'acrobate s'élance en sautant.	Il réalise un saut périlleux.
22	L'urbaniste propose plusieurs projets d'aménagement.	Il a absolument besoin d'un contrat.

Table S2. List of sentences used in experiments 1 and 2. These sentences correspond to the conditions Masculine and Gender-unmarked. In the Feminine condition, the second sentence began with *elle* [she] instead of *il* [he]. In the contracted double form condition, the first sentence began with *un·e* [a$_{MASC\cdot FEM}$] instead of *l'* [the$_{unmarked}$]

2 STATISTICAL MODELS AND RESULTS

In each of the two experiment, two statistical models were fitted to the (zscored) reaction time data and to the percent correct scores. The hierarchy of priors associated with each parameters in the models is summarized in Figure S2. Figures S4 and S5 present the posterior distributions of all parameters estimated in each statistical model.

2.1 Sensitivity analysis

At the suggestion of an anonymous reviewer, a post-hoc sensitivity analysis was carried out in order to measure which effect size could be detected using the sample size and statistical approach followed in the present study, and whether the prior distributions in the model may bias the estimates.

For this purpose, we simulated new sets of data for a range of values of a given parameter and checked whether our statistical analysis was able to retrieve this true parameter value. As the sensitivity analysis is very time consuming, we conducted it only on two parameters of interest in experiment 2: β_{gender} and $\beta_{gender\times condition}$ for the reaction-time model.

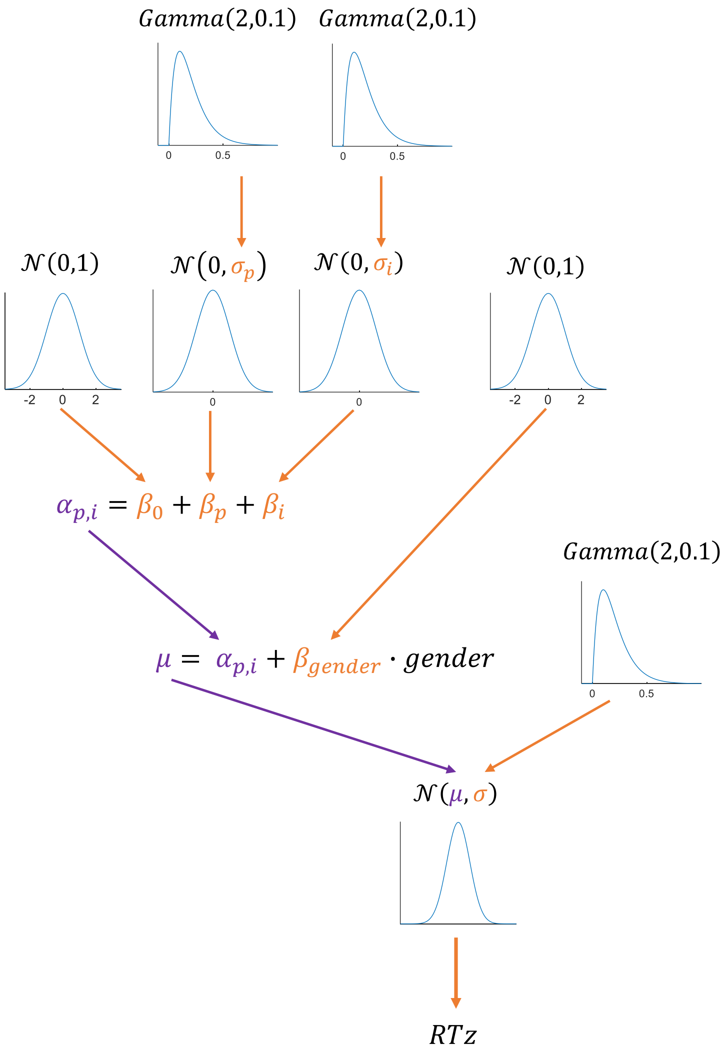

Figure S2. Schematic diagram of the Bayesian hierarchical model fitted to the reaction-time data from Experiment 1. Arrows illustrate the chain of dependencies between parameters and prior distributions. Orange arrows denote stochastic dependencies ("X is drawn from distribution Y"). Indigo arrows correspond to deterministic dependencies. Parameters estimated within the model are indicated in orange.

Forty plausible β parameter values were considered (from -0.2 to +0.2) for each of the two parameters of interest and 2×40 new sets of data were generated, assuming the GLM structure described in the article. For the sake of simplicity, the hierarchical structure of the data (by item and by participant) was not considered. For each simulation, all β parameters were chosen to be equal to the point estimates measured experimentally, except for the parameter of interest which was replaced by one of its "artificial" values. Then, the exact same statistical analysis as described in the article was conducted on these simulated data. The estimated β value was reported, together with its credible interval, as a function of the true β value (see Fig. S3).

This analysis indicated that, assuming that the underlying statistical model is true, our statistical analysis is not able to conclude that a parameter is different from zero for an approximate range of $-0.12 < \beta_{gender} < 0.12$ and $-0.22 < \beta_{gender \times condition} < 0.22$. Furthermore, the weak prior distributions used in the model does not appear to bias the estimates.

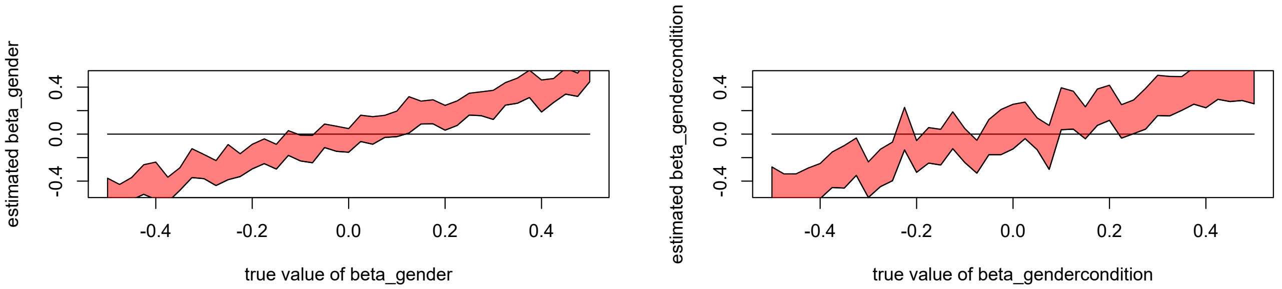

Figure S3. Estimated 95% credible interval for the parameters β_{gender} (left panel) and $\beta_{gender \times condition}$ (right panel), plotted as a function of the true value of the parameter.

REFERENCES

Misersky, J., Gygax, P. M., Canal, P., Gabriel, U., Garnham, A., Braun, F., et al. (2014). Norms on the gender perception of role nouns in Czech, English, French, German, Italian, Norwegian, and Slovak. *Behavior Research Methods* 46, 841–871. doi:10.3758/s13428-013-0409-z

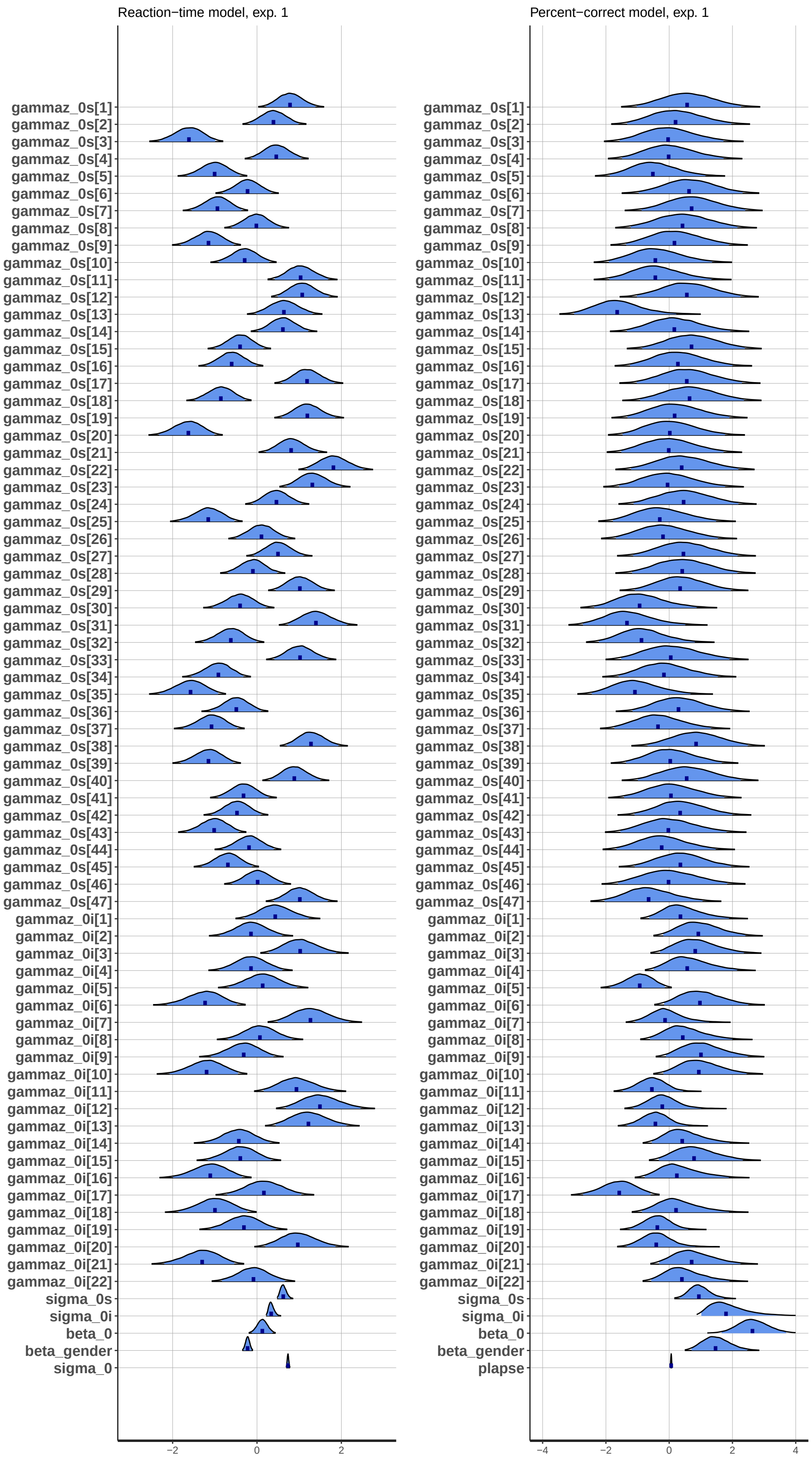

Figure S4. Posterior distributions of all parameters for the two models considered in experiment 1 (left: reaction-time model, right: percent-correct model). The 95% credible interval for each estimate is indicated in blue.

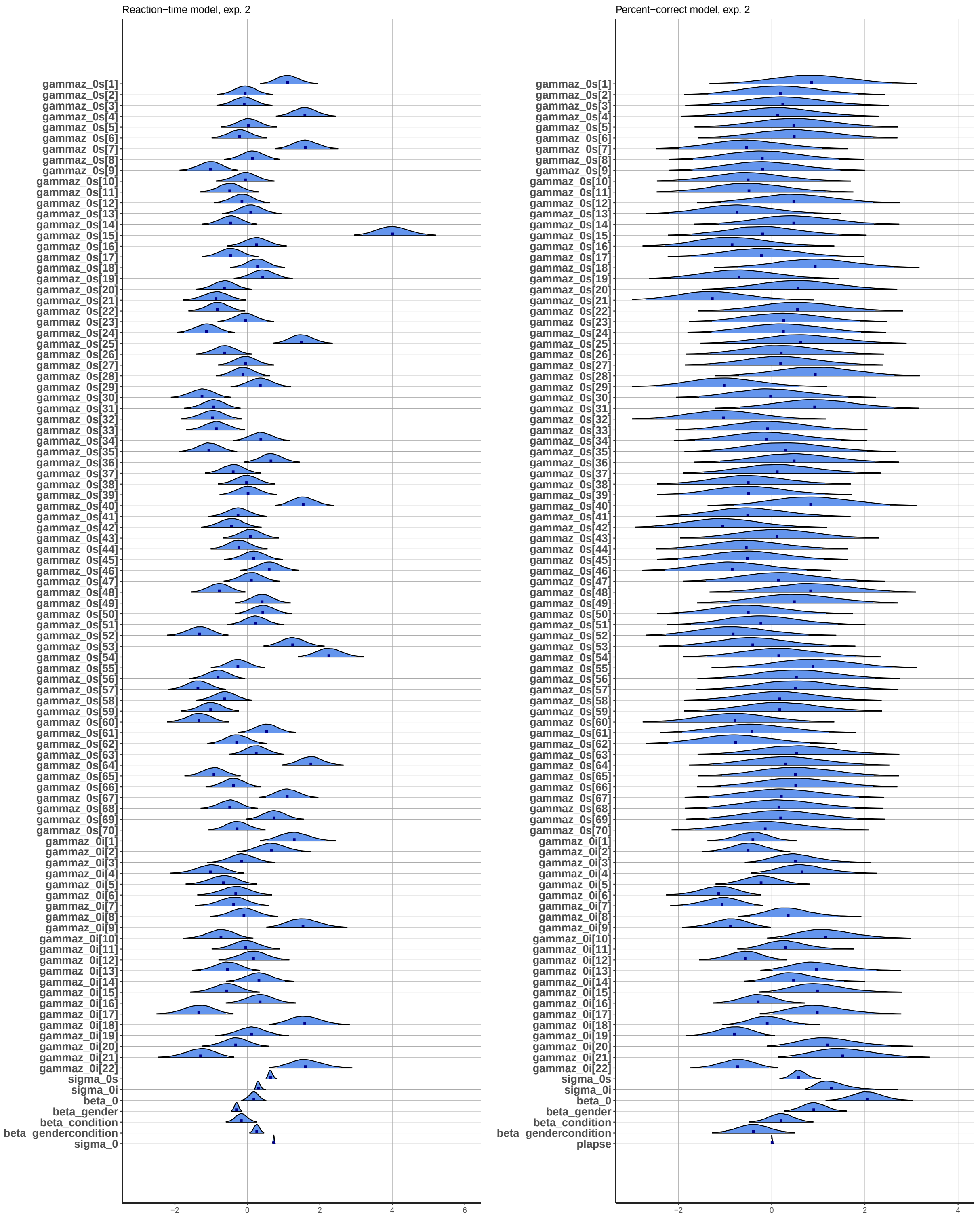

Figure S5. Posterior distributions of all parameters for the two models considered in experiment 2 (left: reaction-time model, right: percent-correct model). The 95% credible interval for each estimate is indicated in blue.
